# Evidence for reciprocal network interactions between injured hearts and cancer

**DOI:** 10.3389/fcvm.2022.929259

**Published:** 2022-07-15

**Authors:** Melisa N. Guler, Nathalie M. Tscheiller, Maria Sabater-Molina, Juan R. Gimeno, Canan G. Nebigil

**Affiliations:** ^1^Faculty of Medicine, University of Campania Luigi Vanvitelli, Caserta, Italy; ^2^University of Strasbourg, INSERM, UMR 1260, Nanoregenerative Medicine, Strasbourg, France; ^3^Fédération de Médecine Translationnelle de l’Université de Strasbourg, Strasbourg, France; ^4^Servicio de Cardiología, Laboratorio de Cardiogenética, Centro de Investigacion Biomédica en Red Enfermedades Cardiovasculares (CIBERCV), Hospital Clínico Universitario Virgen de la Arrixaca-IMIB, Murcia, Spain

**Keywords:** cardiotoxicity, cancer, heart failure, risk factors, mechanism, bilateral interaction, secretoms, inflammation

## Abstract

Heart failure (HF) and cancer are responsible for 50% of all deaths in middle-aged people. These diseases are tightly linked, which is supported by recent epidemiological studies and case control studies, demonstrating that HF patients have a higher risk to develop cancer such as lung and breast cancer. For HF patients, a one-size-fits-all clinical management strategy is not effective and patient management represents a major economical and clinical burden. Anti-cancer treatments-mediated cardiotoxicity, leading to HF have been extensively studied. However, recent studies showed that even before the initiation of cancer therapy, cancer patients presented impairments in the cardiovascular functions and exercise capacity. Thus, the optimal cardioprotective and surveillance strategies should be applied to cancer patients with pre-existing HF. Recently, preclinical studies addressed the hypothesis that there is bilateral interaction between cardiac injury and cancer development. Understanding of molecular mechanisms of HF-cancer interaction can define the profiles of bilateral signaling networks, and identify the disease-specific biomarkers and possibly therapeutic targets. Here we discuss the shared pathological events, and some treatments of cancer- and HF-mediated risk incidence. Finally, we address the evidences on bilateral connection between cardiac injury (HF and early cardiac remodeling) and cancer through secreted factors (secretoms).

## Introduction

### Patients with cardiovascular disease have a higher risk of developing cancer

Heart failure (HF) and cancer are tightly linked (1, 2) which is supported by recent studies on epidemiological cohort and case-control, research synopsis and meta-analyses. An increased cancer risk in HF patients was shown by the international cohorts such as America (3–5), Denmark (6), Japan (7), and Korea (8). These studies demonstrate that HF patients have a higher risk to develop cancer, independently of age (9–11). Furthermore, women with HF are at higher risk than men, indicating that gender is an important factor (3). The most common types of cancer in HF patients below age 55 are colorectal (21%), lung (18%), gastrointestinal (20%); prostate (16%) (6).

### Patient with cancers have higher risk of dying from heart disease and stroke

Cancer patients can develop cardiovascular diseases (CVD) mainly for the three reasons: (1) the anticancer drugs can have direct adverse effect on cardiovascular system (2), soluble factors (secretoms) such as chemokines, hormones, and vesicles released from tumor cells can damage the cardiac cells as a paracrine manner (3), cancer itself or anticancer drugs induce cachexia that leads to cardiac dysfunction (12). As a consequence, approximately 20–30% of cancer patients die from cardiovascular dysfunctions, regardless of the time passed after cancer diagnosis (13). Indeed, 50% of patients with breast, prostate, endometrial, and thyroid cancer die because of CVD (14, 15). In the most aggressive cancer cases such as cancers of the lung, liver, brain, stomach, gallbladder, pancreas, esophagus, ovary, and multiple myeloma, patients die primarily due to cancer (16). However, the CVD -related mortality were higher among the survivals with cancer of bladder (19% of patients), larynx (17%), prostate (17%), uterus (16%), bowel (14%), and breast (12%).

Sturgeon et al. using databases of the Surveillance, Epidemiology and End Results (SEER) found that among the 3,234,256 cancer patients with 28 different types of cancer, 38% of mortality was due to cancer, whereas 11% mortality was from CVDs including hypertension, cerebrovascular disease, arterial diseases, and cardiac ischemia (17). More interestingly, the highest mortality among the younger cancer patients (< 35 years old) 1 year after the cancer diagnosis was due to CVD.

Co-occurrence of both diseases causes a major clinical burden and has a strong outcome on the quality of life and survival rates (10, 18). Early diagnosis and better understanding of bilateral interaction between HF and cancer is critical for an optimal treatment and management strategies, because a one-size-fits-all treatment approach is ineffective for these patients (7, 8).

Here we outline the known common mechanisms and preclinical studies emphasizing the interactions to help mechanistic understanding that may impact on identifying biomarkers and innovative therapeutic strategies targeting both diseases simultaneously.

## Common mechanisms involved in tumor growth and heart failure

**FIGURE 1:**
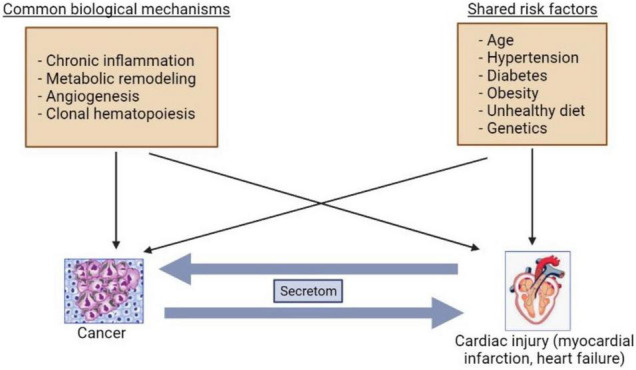
Bilateral interactions between cancer and heart failure. The common mechanisms and risk factors are involved in cancer and heart failure development. The secretoms can be involved in communications between cancer and heart failure.

CVD and cancer share common risk factors such as smoking, aging, genetic predisposition, obesity, and diabetes mellitus (9). Indeed, cardiac regeneration and diseases are reminiscent of processes of tumor development (19). A growing body of studies have suggested that several mechanisms can be involved in development of both HF and cancer such as inflammation, metabolic remodeling, clonal hematopoiesis, angiogenesis, the extracellular matrix (ECM), and stromal cells activations (20, 21) (Figure 1).

### Inflammation

Any changes in homeostasis by stresses, tissue damage, infection, metabolic alterations induces low-grade inflammation to achieve wound healing and tissue regeneration, and to prevent loss of tissue function (22). However, maladaptive chronic inflammation leads to progressive myocardial injury, development of vascular dysfunction, and reduces cardiac tissue survival. The cytokines chemokines, and lipid mediators are involved in inflammatory signaling. Accordingly, high circulating levels of pro-inflammatory cytokines [e.g., interleukin (IL)-1β, and IL-6] have been found in acute and chronic decompensated HF (23). Many cohort studies (> 50) have showed that both high-sensitive CRP (C-reactive protein) and IL-6 can predict development of myocardial infarction (MI) and stroke (24, 25). However, chronic inflammation predisposes to the development of cancer and affects the tumorigenesis and tumor-permissive state by promoting proinflammatory cytokines and chemokines (26). For example, IL-1β and IL-6 have also been reported as key players in development of cancer (27). Specifically, in the solid malignancies, the infiltration of immune cells and the production of pro-inflammatory mediators play key roles for malignant transformation, via epithelial to mesenchymal transition, and metastasis (28).

In the Whitehall II study cohort, Ridker demonstrated that low level of systemic inflammation detected by increased levels of CRP and IL-6 was associated with prediction of cardiovascular and cancer-related mortality in midlife patients (24). Anti-Inflammatory Thrombosis Outcome Study (CANTOS) has also confirmed this study showing that canakinumab an IL-1β-targeting antibody has beneficial effects on cardiovascular events (29). Moreover, canakinumab significantly decreased incident of mortality in patients with lung cancer.

The role of other inflammatory mediators eicosanoids, such as prostanoids (prostaglandins and prostacyclines) in cancer and CVD has not been extensively investigated yet. For example, prostacyclin has been used to treat pulmonary arterial hypertension (30). Unfortunately, in this condition, the pulmonary cancer incidence has not been studied. In contrary, in mice model prostacyclin prevents lung cancer (31). Unlike prostacyclin, prostaglandin E_2_ promotes cancer initiation and lung cancer migration (32) and activates cardiac maladaptive remodeling (33).

All together these studies show that inflammation is one of the shared mechanisms of both HF and cancer. The important question is whether diminishing inflammation can reduce the development rate of CVD and cancer. Use of anti-inflammatory drugs (e.g., low-dose methotrexate, colchicine, and canakinumab) in the large clinical trials should answer this question.

### Metabolic remodeling

The healthy tissues can derive energy from various circulating substrates. However, metabolic alterations due to accumulation of toxic intermediates and utilization of unbalanced substrates can alter the cardiac cell homeostasis and cancer growth. Indeed, recent studies have shown that onco-metabolic dysregulation can promote cardiac dysfunction (34).

Metabolic reprogramming occurs as an adaptive event in both cancer (35) and cardiac cells (36) in response to pathophysiological insult and stress, indicating that both cells share the same metabolic pathways. In HF and cancer, glucose oxidation and glycolysis are central metabolic pathways to generate energy in the form of adenosine triphosphate (ATP) (37, 38). Cancer cells are dependent on aerobic glycolysis that facilitates the incorporation of nutrients into biomass such as nucleotides, amino acids, and lipids to maintain cancer cell proliferation. Aerobic glycolysis is also needed for the adaptive hypertrophy in cardiomyocytes (39). In cancer cells glutamine is the essential carbon source for aspartate synthesis (40). However, in the damaged heart glycolysis and glucose oxidation are predominant over fatty acid oxidation and are required for pentose phosphate pathway (41).

Because of the upregulated glucose utilization in many solid tumors (42), as well as in the failing heart (43), glucose transporter 1 (GLUT1) becomes an important target for the treatment of cancer and HF. Additionally, it has been shown that sodium glucose co-transporter 2 (SGLT2) inhibition has beneficial effects on the heart as well as in pancreatic and prostate cancers. Thus, inhibition of glucose transports may prevent cardiac hypertrophy (44) and reduce cancer growth (45). However, more extensive researches are required to define their beneficially effects during development and progression of different cancer types and CVDs, before their clinical applications.

*De novo* lipogenesis leading to lipo-expediency has also emerged as common mechanisms of HF and cancer. Upregulation of fatty acid synthase (FAS), a key enzyme of *de novo* lipogenesis, has been found in both cancers (46) and HF patients (47). FAS inhibitors have anti-neoplastic properties in solid cancers and represents a potential therapeutic target for HF.

Changes in mitochondrial metabolism is also a common mechanism of HF and cancer development. Inhibition of mitochondrial electron transport chain (ETC) complex I can decrease mitochondrial ATP delivery (48), thereby limiting glucose availability. Additional to ETC inhibitory effects, metformin has been shown to reduce plasma levels of insulin and insulin-like growth factor 1 (IGF-1) to limit glucose availability in the glycolysis-dependent cancer cells (49). In cancer cells (e.g., pancreas cancer), an increased utilization of glutamine, a major substrate for respiration, is required in supporting macromolecule synthesis and maintain the redox homeostasis to contribute cancer growth (50). Thus, inhibitors of glutaminase, a catalyzer of the conversion of glutamine to glutamate, regulate redox balance, and autophagy, induce apoptosis *via* mTOR signaling, and promote growth arrest. On the other hand, oxidative stress upregulates glutaminase 1 and promotes glutaminolysis in the heart. Inhibition of glutaminase improves maladaptive cardiac remodeling and improves sustain activation of autophagy-mediated reduced cardiac contractility (51).

The common maladaptive metabolism pathways in cancer and HF can provide opportunities to the discovery of new biomarkers, and development of juncture strategies and therapies to battle these diseases, and may anticipate to the metabolic phenotyping of diseases in the precision medicine in the field of cardio-oncology.

### Angiogenesis

Angiogenesis is involved in the pathophysiology of both development of HF (52) and cancer (53). Angiogenesis is crucial for tumor growth and metastasis (54), whereas vascular refraction in the maladaptive sustained pressure overload contributes to the transition from compensated hypertrophy to HF (55). Because of increased oxygen demand in tumor or ischemic hearts, hypoxia upregulates HIF1a that promotes expression of angiogenic factors, such as vascular endothelial growth factor (VEGF) (56), angiopoietin-1 and -2 (57), and prokineticin (58, 59) to stimulate microvascular expansion.

The pharmacological or genetic inhibition of VEGF, and other key angiogenic signaling pathways accelerate the transition from adaptive cardiac remodeling to HF (60), while anti-angiogenic therapy beneficial to cancer (e.g., metastatic colon cancer, non-small cell lung cancer, breast cancer). However, cancer cells develop adaptive resistance to the anti-angiogenic therapy as well as severe cardiotoxicity, leading to development of ischemic CVD and HF (61). Thus, angiogenesis delineates an auspicious substrate for both cancer and HF.

### Clonal hematopoiesis

Genetic assets leading to hematologic malignancies such as somatic mutations in hematopoietic stem cells are the potent risk factors for CVD and cancer (62). The mutation on the genes encode for key epigenetic regulators of hematopoiesis leads to the abnormal expansion of clonally derived hematopoietic stem cells (63). A higher frequency of accumulation of hematopoietic mutations in DNA methyltransferase 3 alpha (DNMT3α), Ten-eleven translocation-2 (*TET2)*, additional sex combs like 1 (ASXL1), Janus kinase 2 (JAK2), and tumor protein 53 (TP53) (64) have been found in individuals with lymphoid or solid tumors who are exposure to genotoxic stress (65). Hematopoietic mutations in *DNMT3a*, *TET2*, and *JAK2*^*V*671*F*^, can accelerate atherosclerosis and the increase risk of CVD by generating a pool of myeloid cells with an augmented proinflammatory profile (66). These mutations are associated with worse outcomes in patients with ischemic HF (67, 68).

Identification of the mechanisms linking somatic mutation-driven clonal hematopoiesis to CVDs is off interest specifically in personalized medicine. There are some questions needs to be addressed such as whether clonal hematopoiesis also contribute to CVD in cancer survivors (69) and whether these mutations can be predictive markers of cardiovascular risk and therapeutic responsiveness.

### Cardiogenetic: Cardiac-associated genetic variant to cancer predisposition

Recent studies have demonstrated that 50% of non-ischemic cardiomyopathies caused by more than gene variants encoding for cytoskeleton, ion channels, nuclear envelope, intercellular junctions sarcolemma and sarcomeric proteins (70). A genetic predisposition to therapy-induced cardiomyopathy has been observed in families with history of hypertrophic, dilated and arrhythmogenic cardiomyopathies (71). Patients with cardiomyopathy or asymptomatic carriers of inherited cardiac diseases have a potential increased risk for cardiotoxicity induced by anticancer treatment (72).

Interestingly, several of these genes associated with familial cardiomyopathies harbor relevant genetic variants in somatic cancer cells. A high prevalence of somatic mutations in Titin (*TTN*), Dystrophin (*DMD*), and Desmoglein 2 (*DSG2*) have been associated with different stages of carcinogenesis process.

The KEGG (Kyoto Encyclopedia of Genes and Genomes terms) analyses have identified the total of 33 genes and 25 links with 17 metabolic pathways that can be implicated in interaction between genetic cardiomyopathies and molecular pathways of cancer. The genes involved in both cardiomyopathy and carcinogenesis include Protein tyrosine phosphatase, non-receptor 11 (*PTPN11*) and LMNA, another 12 genes from sarcomeric (thin and thick filament), desmosomal (*PKG/JUP*), metabolic (*PRKAG2, LAMP2, GLA*), and calcium handling (*PLN*) (73). For example, the RAS family of small Guanosine Triphosphate (GTP)-binding proteins (G proteins) plays a key role in intracellular signal transduction required for normal cardiac growth, development of hypertrophic cardiomyopathy and HF as well as cancer (74). RASopathies are single-gene inheritance disorder caused by germline mutations in genes that encode constituents or regulators of the RAS/mitogen-activated protein kinase (MAPK) pathway. RASopathies are accompanied with the higher risk of hematologic or solid cancer and congenital CVD (75). For example, hypertrophic cardiomyopathy development during childhood can be triggered by genetic mutations in *PTPN11*, KRAS, Son of sevenless homolog 1 (*SOS1*), a RAS effector (*RAF1*), genes which are also involved in cancer developments (73).

The signaling pathway of wingless-related integration site (Wnt) controls proliferation and differentiation processes in different types of cancer. Indeed, in patients with desmosomal mutations on the Wnt pathway exhibit the histological fibro-adipose differentiation, a characteristic of arrhythmogenic cardiomyocardiopathy (76).

Additional mechanistic studies on damaging gene variants in CVD and cancer can unravel prognostic biomarkers and new treatment strategies for both disorders.

## Cardiovascular drug: Promoter or suppressor of cancer incidence?

**FIGURE 2:**
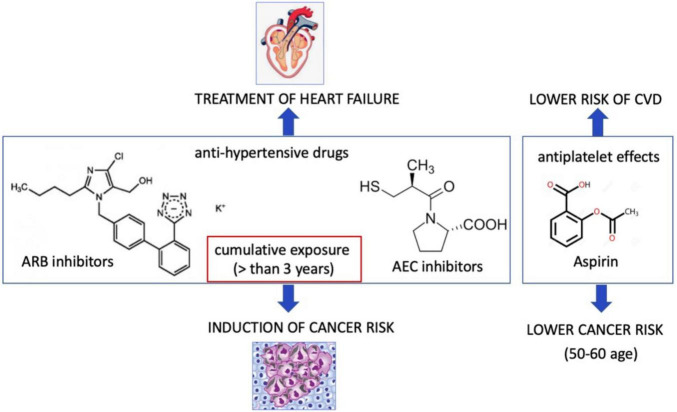
Cardiovascular drugs may promote or suppress cancer incidence. Angiotensin receptor II blocker that are used for treatment of hypertension at the cumulative exposure more than 3 years may induce cancer risk. Aspirin may lower cancer risk.

HF results in the hyperactivity of neurohormonal systems, including the renin–angiotensin–aldosterone (RAA) system and the sympathetic nervous system (77). Interestingly, noradrenaline and angiotensin II also play an important role in modulation of tumor microenvironment and tumor development (78). Contrarily, several studies demonstrated that patients treated with angiotensin-converting enzyme (ACE) inhibitors but not angiotensin receptor blocker (ARB) for more than 5 years have a higher lung cancer incidence (79). However, subgroup analysis has demonstrated a significant association between ARB and cancers in male genital organs (80). In contrary, the recent study demonstrated an association between the ARB and decreased risk of overall cancer and several site-specific cancers (81). Patients treated with hydrochlorothiazide, a diuretic drug, had a higher prevalence to have basal and squamous cell carcinoma (82). A large meta-analysis on hypertensive patients treated with all types of anti-hypertensive drugs (ARBs, ACEi, β-blockers, diuretics, and calcium channel blockers), demonstrated a 5.0–10.0% increase in the risk of cancer or cancer-related death (83). ARB use in patients with type 2 diabetes demonstrated a negative association for losartan (ARB), but a positive association for candesartan and telmisartan with the overall occurrence of cancer (84). Despite some contradictory conclusions about ARB mediated cancer risk, a recent study considering the exposure-risk relationship and using data from all 15 trials and randomized controlled trials has resulted in a very significant correlation between the degree of cumulative exposure (greater than 3 years) to ARBs and risk of all cancers especially lung cancers (85). In this study, patients with lower cumulative exposure to ARBs did not exhibit an increased risk of all cancers combined or lung cancer, explaining the heterogeneity in the results of randomized trials, due to terms of cumulative exposure to ARB (Figure 2).

The other cardiovascular drug is aspirin that leads to antiplatelet effects *via* inhibition of platelet cyclooxygenase (COX-1) and blockade of the production of thromboxane A2. Aspirin uses both cyclooxygenase-dependent and cyclooxygenase-independent mechanisms in cancer (86). Low-dose aspirin did not lower the cancer incidence in a low or medium CVD risk population (87, 88). The U.S. Preventive Services Task Force (USPSTF) recommended that use of low dose aspirin can reduce risk of CVDs and colorectal cancer among the people at the age of 50–60 (89). Thus, these benefit effect of aspirin may not translate to older adults (90). Currently, the mechanism of beneficial effects of aspirin is not known. The appropriate pre-clinical models are emerging to discover the molecular and cellular mechanism aspirin and ARBs in patient with cancer and CVD (Figure 2).

## Pre-clinical models to study bilateral interaction between heart failure and cancer

Despite most of epidemiological studies showed high prevalence of cancer development in HF patients, they cannot prove direct interconnection between HF and cancer. There are some preclinical studies aimed to explore the bilateral relationship between HF and cancer development.

### Cancer to heart failure

**FIGURE 3:**
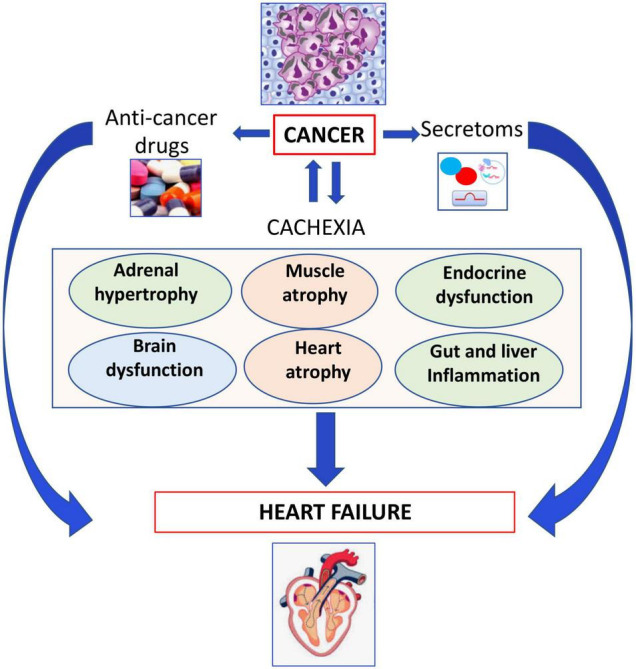
Role of cachexia in development of heart failure. Cancer or anticancer drug-mediated cachexia may induce several organ dysfunctions leading to heart failure.

Cancer or anti-cancer drug-mediated cachexia and cancer-secretoms, adverse effects of anticancer drugs may induce several organ dysfunctions and HF as outlined in Figure 3.

#### Cancer cachexia promotes cardiac atrophy

Cachexia is defined as a state of involuntary weight loss. The symptoms of cancer cachexia such as fatigue, shortness of breath, and impaired exercise capacity are also symptoms of HF. Cachexia prompts metabolic changes in the metabolisms of lipids and proteins, leading to a negative nitrogen balance and reduction of the protein levels, causing insulin resistance, and anemia. Cachexia affects approximately 32% of cancer patients within half a year at the time of diagnosis, and causes one third of cancer deaths (91). Patients with pancreatic, gastro-oesophageal, lung, head and neck and colorectal cancers often have cachexia with a prevalence between 40 and 70% (92). It also co-occurs with metastasis in 80% of late-stage cancer patients (93).

The mechanism of cancer cachexia is not known, but Indeed, cancer patients, with and without cachexia exhibit the high levels of serum brain natriuretic peptide (BNP), renin and aldosterone (94). These altered levels of hormones increase sensitivity to infections, and decrease responsiveness to both chemotherapy and radiation treatment. They also cause loss of muscle protein that promotes muscle weakness and fatigue, and cardiac or respiratory failure (95). Moreover, the heart becomes atrophic in cancer patients. There are also several direct experimental evidence showing that cancer-mediated muscle wasting and cachexia (96) can be key players of cancer-related death. Cancer itself can result in cardiac atrophy as well (97).

Cancer cachexia-mediated a waste of skeletal muscle and adipose tissue has been observed more severely in men more than women. The mechanisms of the sex differences in cancer cachexia have been experimentally studied by Cosper and Leinwand (98). In this study the tumor bearing (CD2F1) mice with injection of Colon-26 adenocarcinoma (C-26) has displayed a rapidly and increasingly loss of cardiac mass during the course of tumor progression. Significant differences in the disease phenotype have also been observed between males and females. For example, male mice exhibit more waste in body weight, skeletal, and cardiac muscle and the higher cardiac dysfunctions than females. The decrease in all myofibrillar proteins, at the expenses of myosin heavy chain (MyHC) in heart is due to autophagy as a main proteolytic pathway. In this study, the estrogen receptor signaling has been shown to protect females against the loss of body weight and cardiac mass. Indeed, it appears that activation of Ca^2+^ dependent atrophy is also involved in wasting in skeletal muscle and heart of male Wistar rat bearing Yoshida AH-130 ascites hepatoma cells for 6 days as a cancer cachexia models (99).

Springer’s group using the rat hepatoma model (AH-130-bearing rats) demonstrated that weight loss affects predominantly skeletal muscle and myocardium associated with left ventricular-dysfunction, fibrotic remodeling, and increased mortality (100). They found that several key anabolic and catabolic pathways were dysregulated in the cachectic hearts. These detrimental effects of the tumor on the heart and on survival can be alleviated by treatment with the β-blocker bisoprolol or the aldosterone antagonist spironolactone (101). Incoherent to this study, Toledo et al. have shown that the administration of a highly potent β2-adrenoceptor-selective agonist, formoterol, to cachectic tumor-bearing rats caused a significant reduction of muscle weight loss, an increase in lean body mass probably due to preventing muscle apoptosis and the increased muscle regeneration (102). The clinical trial phase 2 has shown *f*ormoterol has beneficial effects in patients with advanced cancer (103). Costelli et al. in AH-130-bearing rats have shown that β2-adrenoceptor agonists, clenbuterol, prevents skeletal muscle waste, however, it has no effect parenchymal organs (104).

Same group has shown that the mechanisms of muscle depletion is due to increased proteasome- and calpain-dependent proteolysis in tumor bearing rats treated with TNF-alpha synthesis inhibitor, or an antiprotozoal drug blocking the IL-6 and TNF-alpha action (105).

A variety of cytokines have also been proposed to trigger cancer-induced cardiac muscle wasting. Zhou X ‘s group has studied involvement of a high affinity activin type 2 receptor (ActRIIBa), that binds to TGF-β family ligands (myostatin, activin, Growth differentiation factor 11), utilizing two animal models; the tumor-bearing mice (colon 26, human G361 melanoma and TOV-21G ovarian carcinoma) and inhibin-knockout mice (106). Indeed, activation of ActRIIB pathway enhances ubiquitination of muscle proteins that are key pathways in muscle wasting. Indeed, inhibition of the ActRIIBa in this study has fully restored the loss of muscle during cancer cachexia, without altering the high levels of the inflammatory cytokine levels.

The emerging question in this area is whether animal models consistent with clinical cancer cachexia and the potential drugs can have beneficial effects in cancer patients (107).

#### Cancer cells derived secreted factors (secretoms) promote cardiac atrophy

Cancer cells release secreted factors (secretoms) result in developing cardiac atrophy and metabolic changes, but the exact signaling pathways in cardiomyocytes are still poorly understood (108). Cancer cells-mediated systemic metabolic alterations may impair cardiac function (39). Alterations in metabolic fueling of the heart as well as metabolic intermediates can alter gene expression, protein function and provoke epigenetic modifications thereby stemming in ventricular remodeling.

Somatic mutations in metabolic regulator genes could be the mechanism of cancer-mediated cardiac dysfunction. For example, somatic mutation in dehydrogenase (IDH1/2) gene causes in a gain-of-function, thereby allowing synthesis of 2-hydroxyglutarate (2-HG) that is structurally similar to α*-*ketoglutarate (α-KG), a key regulatory enzyme of cellular energy metabolism and an intermediate of the tricarboxylic acid (TCA) Krebs cycle (109). An increased circulating levels of 2-HG triggers dilated cardiomyopathy and contractile defects by impairing α-KG pathway, and leading to mitochondrial damage and myocardial glycogen accumulation (39).

More studies are required to identify novel factors that can be important to stratify the risk of development of CVD in cancer patients.

#### Anti-cancer-induced cardiotoxicity

**FIGURE 4:**
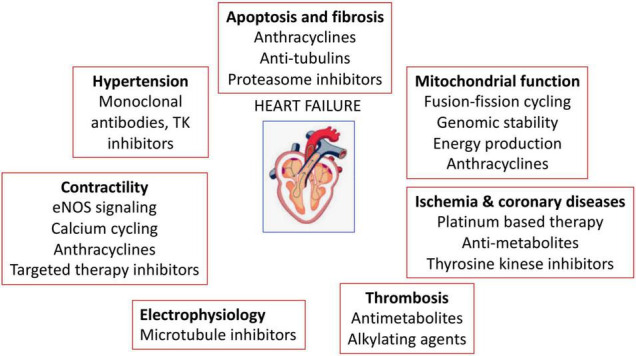
Anticancer drugs-mediated cardiac adverse effects. Anticancer drugs can induce hypertension, cardiovascular damage, ischemia and coronary diseases, thrombosis, contractility defects and arrhythmias.

The anticancer-drug induced cardiotoxicity has been widely studied, which can occur during, shortly after, or many years after cancer therapy. Cardiotoxicity can range from subclinical myocardial dysfunction to irreversible HF. Thus, in the long-term, the risk of death can be due to cardiovascular dysfunctions rather than tumor recurrence (39–41). The cancer therapy related-cardiovascular complications are listed in Figure 4 and Table 1 (110).

**TABLE 1:** Some of the anticancer drugs-mediated adverse effects in cardiovascular system.

Anticancer drugs	Clinical manifestation
Anthracyclines (e.g., doxorubicin)	LV-dysfunction, contractile defects, ischemia, thromboembolism
Targeted therapy/TK inhibitors (e.g., bevacizumab, sorafenib, nilotinib) and anti-angiogenic therapy (e.g., VEGF inhibitor)	Hypertension, Bradycardia, QT-prolongation, contractile defects, Ischemia, and coronary diseases, venous thromboembolism
Immune check point inhibitors (e.g., ipilimumab, nivolumab)	Myocarditis/pericarditis

**FIGURE 5:**
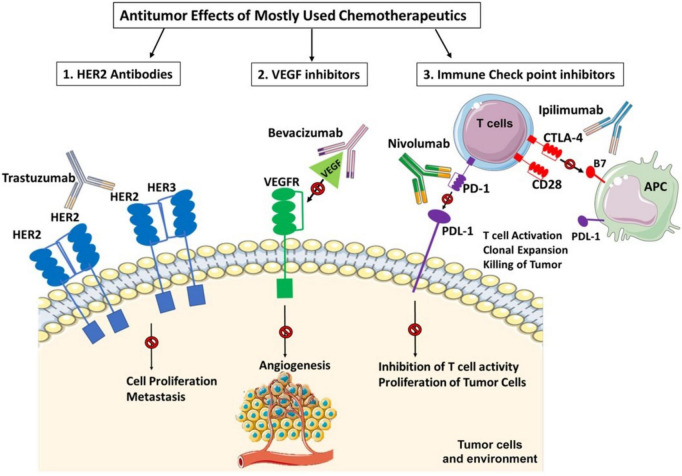
Three widely used anticancer drug mediated cytotoxicity in cancer and heart. Clinically used targeted therapies/tyrosine kinase (TK) inhibitors mainly focuses on inactivation of heregulin receptor (HER2) or vascular endothelial factor (VEGF) signaling in cancers. HER2 signaling is important in heart and cancer. HER2 activation by its ligand heregulin stimulates proliferation pathway in the tumor cells. The inhibition of HER2 by its antibody, trastuzumab, in cancer cells blocks the cancer proliferation but in heart it blocks an important survival pathway in heart. Binding of VEGF to its receptors in endothelial cells activates angiogenesis. Inhibition of VEGF by its antibody, bevacizumab, can destroy the tumor angiogenesis and bring the tumor to avascular stage, whereas it can be detrimental in the heart due to reduced systemic angiogenesis. Indeed, the immunotherapy targeting cytotoxic T-lymphocyte antigen 4 (CTLA-4). anti-programmed cell death 1 (PD1) or its receptor (PDL-1) relies on cancer destruction through the activation of the host immune system. However, PD-L1 is also expressed in the non-immune cells to maintain self-tolerance. Nivolumab and Ipilimumab activate T cells and promotes T cell clonal expansion to kill tumor cells. However activated T cells also recognize shared antigens and destroy cardiomyocytes as well.

One of the important reasons for cardiotoxicity is that the anticancer drugs use similar pathways and targets in both cancer and heart cells to exert their cytotoxic effects as described in Figure 5. The anthracycline group of anticancer drugs use the same signaling pathways to induce cytotoxicity in both cancer and cardiac cells, leading to HF-related morbidity and mortality. The mechanism of anthracycline-mediated cardiotoxicity has been widely studied and recently reviewed by Nebigil and Désaubry (111). Targeted therapies such as tyrosine kinase inhibitors have also adverse effects on cardiovascular system. For example, heregulin receptor, HER2, express in both cancer and cardiac cells. Inhibition of HER2 by antibodies blocks the cancer cell proliferation, but it also blocks an important survival pathway in heart (112). Binding of VEGF to its receptors in endothelial cells activates angiogenesis. VEGF inhibitors can destroy the tumor angiogenesis and bring the tumor to avascular stage, whereas it can be detrimental in the heart due to reduced systemic angiogenesis (113). Checkpoint inhibitors induce T-cell activation. Monoclonal antibodies that are used to block immune inhibitory checkpoints target cytotoxic T-lymphocyte antigen 4 (CTLA-4 (ipilimumab, nivolumab, pembrolizumab), or anti-programmed cell death 1 (PD1) (atezolizumab, avelumab, durvalumab). The mechanisms of the adverse effects of immune checkpoints inhibitors are (1) release of cytolytic molecules (e.g., tumor necrosis factor-α, granzyme B, interferon-γ) that kill tumor cell and promote autoimmune lymphocytic myocarditis, (2) the PD-L1 and CTLA-4 are also expressed in heart and tumors and can share antigens that recognize by the same T-cell clones. Thus, activated T cells attack not only on tumor cells but also on cardiomyocytes, and destroys hearts (114).

Thus, the paracrine effect of anticancer drugs-mediated cardiotoxicity remains to be widely investigated.

### Heart failure to cancer

#### Myocardial infarction mediated secretoms and innate immune system promotes cancer development

Kitsis et al. investigated the interaction between HF and cancer by creating MI-induced HF in a precancerous murine model, adenomatous polyposis coli *(APC)^–/–^*mouse strain (115). These mice carried a non-sense mutation in *APC* leading to persistence of beta-catenin that induces spontaneous intestinal adenoma formation (1). The experimental HF on those mice resulted in increased tumor formation and tumor growth, 6 weeks after the MI procedure. To rule out the possibility that hemodynamic impairments lead to tumor growth, the failing heart has been transplanted into these precancer mice as a heterotopic murine (HFTx) heart model. Indeed, this model further proved that HF can contribute to tumor formation and progression. The candidate secreted molecules of HF were then identified based on meta-analyses from databases of proteins secreted from myocardium, and allied to the proteins previously associated with new-onset colorectal cancer. Five potential secreted proteins have been discovered, namely: α-1-antitrypsin (SerpinA1), α-1-antichymotrypsin (SerpinA3), fibronectin, ceruloplasmin, and paraoxonase 1. Indeed, serpinA3 promoted proliferation of the colon cancer cell that was associated with Akt-S6 phosphorylation. Moreover, Kitsis et al. found that the increased levels of serpinA3 and A1, fibronectin, ceruloplasmin, and paraoxonase 1 in patients with chronic HF (115).

Further studies need to establish whether (1) these secretoms can be used as cancer biomarkers to stratify the cancer risk in HF patients, (2) these secretoms can promote tumor formation as well as tumor progression.

The possible role of the immune system in HF to cancer has also been off great interest. Indeed, monocytes and monocyte-derived macrophages play key roles in cancer, such as promoting angiogenesis, tumor cell proliferation, migration, invasion as well as tumor immune evasion. On the other hand, post MI provokes sympathetic signaling such β3 adrenergic stimulation together with IL-1β) release, thereby activating leukocyte progenitors in the bone marrow, and monocytes, in the circulation (116). Recently using 2 mice model of breast cancers: C57BL/6J female mice orthotopically implanted the murine mammary cancer cell line (E0771) and genetically engineered mouse breast cancer model (MMTV-PyMT), Koelwyn et al. demonstrated that MI is an acute pathologic stimulus that induces the innate immune system, to accelerate breast cancer growth and metastasis as well as cancer-associated mortality in mice and humans (116). More specifically, MI epigenetically reprograms Ly6Chi monocytes to give rise to an immunosuppressive phenotype in the bone marrow. In parallel, MI increases circulating levels of Ly6Chi monocytes and recruitment of these monocytes to tumors. Furthermore, depletion of these cells abolishes MI-induced tumor growth. Interestingly, epidemiological studies showed that early stage breast cancer patients who had cardiovascular disorders before the treatment with the chemotherapeutics had increased risk of reappearance of tumor and cancer-specific death.

These preclinical and clinical results are important to understand host comorbidities and their impact on cancer progression.

#### Transverse aortic constriction mouse model of pathological hypertrophic cardiomyopathy promotes cancer

Avraham et al. investigated whether pathologic hypertrophic cardiomyopathy can alter tumor growth and progression, using transverse aortic constriction (TAC) as a mice model of pressure overload–induced cardiac hypertrophy in 2 type mouse syngeneic tumor models: a breast orthotopic cancer model (mouse mammary tumor virus–polyomavirus middle T antigen) and a lung cancer model (Lewis lung carcinoma) (117). This experimental TAC resulted in increased tumor growth and metastatic colonization. After TAC, the tumor implanted mice had a cardiac hypertrophy, and increased volume of tumors. Avraham et al. also investigated the role of host immune system in TAC-associated an increase in tumor growth, using immunodeficient NOD/SCID mice, which lack T and B lymphocytes, with reduced natural killer cell function. Indeed, increased tumor growth after TAC was also observed, eliminating the possible role of the adaptive immune system.

Transcriptomic profiling of the mice TAC-hearts revealed a number of upregulated secretoms known as pro-tumorigenic, such as connective tissue growth factor and periostin. Indeed, depletion of periostatin from the sera of TAC-mice abolished the proliferation of polyomavirus middle T antigen cells. Exogenous addition of periostin increased polyomavirus middle T antigen and Lewis lung carcinoma cell proliferation *in vitro*, showing that periostin in sera of the TAC-operated mice plays key role in cancer cell proliferation. Unfortunately, there was no studies showing that periostin inhibition *in vivo* reduces the tumor growth. Future *in vivo* studies are necessary to determine whether periostin and other secreted factors, promote hypertrophic cardiac remodeling-mediated tumorigenesis.

The mechanisms by which these secretoms exert tumorigenic effect need to be studied. These preclinical studies may open an avenue for the discovery of the heart-specific tumor markers and new therapeutic options.

## Perspective

Recent clinical studies have shown that the mortality of certain cancer patients results from CVD such as HF, hypertension aneurysm of blood vessels and stroke (118). The risk of CVD mortality occurs during an acute phase (early risk) and a chronic phase (late risk) (119). Accordingly, cancer survivors who were diagnosed cancer before the age of 55 years displayed ten-fold higher CV-dependent mortality compare to the general population (118). On the other hand, the cancer risk incidence is high in the patients with HF (120).

Several limitations of the clinical studies showing a link between cancer and HF should be considered as well. For example, in some clinical studies the type of treatments the patients received have not been known to evaluate whether these therapies have adverse effects. Some of these studies have lack of information on co-illnesses and risk factors (e.g., smoking, alcohol consumption, obesity). The socioeconomic status should also be taken in account in these epidemiological studies. Moreover, these studies have been mostly performed on western population, therefore the percentage of risks may range in the different populations.

Preclinical studies are important to unravel the molecular pathways and targets involved in bilateral interaction between CVD and cancer. Cardiac- and cancer-secretoms have potential utility as biomarkers that can be used to identify risks in patient with HF in terms of cancer risk or vice versa. These data will exemplify the importance of understanding the development of comorbidities and help to implementation of strategies for better management of these patients and identify the cardiovascular or cancer-specific mortality.

Nevertheless, the cardio-oncology care should assess regularly the risk of development cancer in HF patients, and CVD in the cancer patients before starting chemotherapy.

## Author contributions

MG and NT prepared the illustration, organized and partially wrote the manuscript. MS-M, JG, and CN wrote and edited the manuscript. All authors contributed to the article and approved the submitted version.

## Conflict of Interest

The authors declare that the research was conducted in the absence of any commercial or financial relationships that could be construed as a potential conflict of interest.

## Publisher’s Note

All claims expressed in this article are solely those of the authors and do not necessarily represent those of their affiliated organizations, or those of the publisher, the editors and the reviewers. Any product that may be evaluated in this article, or claim that may be made by its manufacturer, is not guaranteed or endorsed by the publisher.
